# A Novel Thoracic Ultrasound Measurement After Congenital Diaphragmatic Hernia Repair Identifies Decreased Diaphragmatic Excursion Associated With Adverse Respiratory and Surgical Outcomes

**DOI:** 10.3389/fped.2021.707052

**Published:** 2021-08-05

**Authors:** James T. Ross, Norah E. Liang, Andrew S. Phelps, Anthony I. Squillaro, Lan T. Vu

**Affiliations:** ^1^Department of Surgery, University of California, San Francisco, San Francisco, CA, United States; ^2^Department of Surgery, Massachusetts General Hospital, Boston, MA, United States; ^3^Department of Radiology, Oregon Health and Science University, Portland, OR, United States; ^4^Division of Pediatric Surgery, University of California, San Francisco, San Francisco, CA, United States

**Keywords:** congenital diaphragmatic hernia, ultrasound, diaphragmatic function, outcomes, thoracic surgery

## Abstract

**Background and Aim:** Congenital diaphragmatic hernia (CDH) is a rare defect often associated with pulmonary hypoplasia and abnormal pulmonary vascular development. Even after successful hernia repair, pulmonary disease may persist into adulthood. Impaired diaphragmatic motility may lead to compromised respiratory function long after index repair. This study investigates whether a novel ultrasound measurement, the diaphragmatic excursion ratio, can be a simple and non-invasive method to evaluate routine diaphragmatic motion after CDH repair, and whether it correlates with adverse surgical and respiratory outcomes.

**Materials and Methods:** A cross-sectional study was conducted in consecutive patients who presented at medium-term follow-up visit between December 2017 and December 2018 after CDH repair at single pediatric hospital. Transthoracic ultrasound was performed with craniocaudal diaphragmatic excursion measured bilaterally during routine breathing. Diaphragmatic excursion ratios (diaphragmatic excursion of repaired vs. unrepaired side) were calculated and retrospectively compared with clinical data including demographics, length of stay, respiratory adjuncts, oral feeding, and need for gastrostomy.

**Results:** Thirty-eight patients (median age at ultrasound, 24 months, interquartile range 11–60) were evaluated. Nine patients underwent primary repair, 29 had non-primary repair (internal oblique muscle flap or mesh patch). Patients with a diaphragmatic excursion ratio below the median (0.54) had longer hospital stays (median 77 vs. 28 days, *p* = 0.0007) more ventilator days (median 16 vs. 9 days, *p* =0.004), and were more likely to have been discharged on oxygen (68 vs. 16%, *p* = 0.001). They were also less likely to be exclusively taking oral feeds at 1-year post-surgery (37 vs. 74%, *p* = 0.02) and more likely to require a gastrostomy tube in the first year of life (74 vs. 21%, *p* = 0.003).

**Conclusions:** Transthoracic ultrasound after CDH repair is practical method to assess diaphragm motion, and decreased diaphragm excursion ratio is associated with worse respiratory outcomes, a longer length of stay, and dependence on gastrostomy tube feeding within 1 year. Further prospective studies may help validate this novel ultrasound measurement and offer prognostic value.

## Introduction

Congenital diaphragmatic hernia (CDH) is a rare congenital defect of the diaphragm that is often associated with abnormal pulmonary development. Pulmonary hypoplasia contributes to significant respiratory morbidity after surgical repair of CDH and obstructive pulmonary disease may persist into adulthood (1). As mechanical forces are critical to normal lung development (2), there has been considerable interest in studying the respiratory mechanics of patients after CDH repair to identify patients at risk of respiratory complications, and to understand the changes in diaphragm function after repair.

CDH defects may be repaired primarily, with the use of an internal oblique muscle flap (IOMF), or with a mesh patch. Primary repair is typically only possible in small defects (3). In contrast, mesh patches have historically been used only in large defects. Either as a result of disease severity or of some feature of the repair, mesh repair is an independent predictor of poor outcomes in patients with CDH (3, 4). As comfort and experience with IOMF has improved, this technique has been used with increasing frequency even in large diaphragmatic defects (5, 6).

Overall, the outcomes after CDH repair have improved markedly in the last 40 years with improved surgical techniques and the development of advanced adjuncts including high frequency oscillatory ventilation (HFOV), inhaled nitric oxide, and extracorporeal membrane oxygenation (ECMO) (7–9). However, long-term studies of patients who have undergone CDH repair as infants demonstrate that respiratory morbidity persists into early adulthood (1, 10, 11). Existing measures of CDH disease severity, including the observed-to-expected Lung area to Head circumference Ratio, are surrogate markers for the size of the diaphragmatic defect and how that affects prenatal lung development (12). However, the impact of surgical repair of CDH on diaphragm and respiratory function is poorly understood, and there are few non-invasive measures by which to evaluate diaphragmatic motion following repair.

Thus, this study was designed to test the practicality and utility of ultrasound to measure diaphragmatic excursion ratios in patients following CDH repair, and to determine whether this newly defined measure is associated with any surgical and respiratory outcomes.

## Materials and Methods

**Figure 1:**
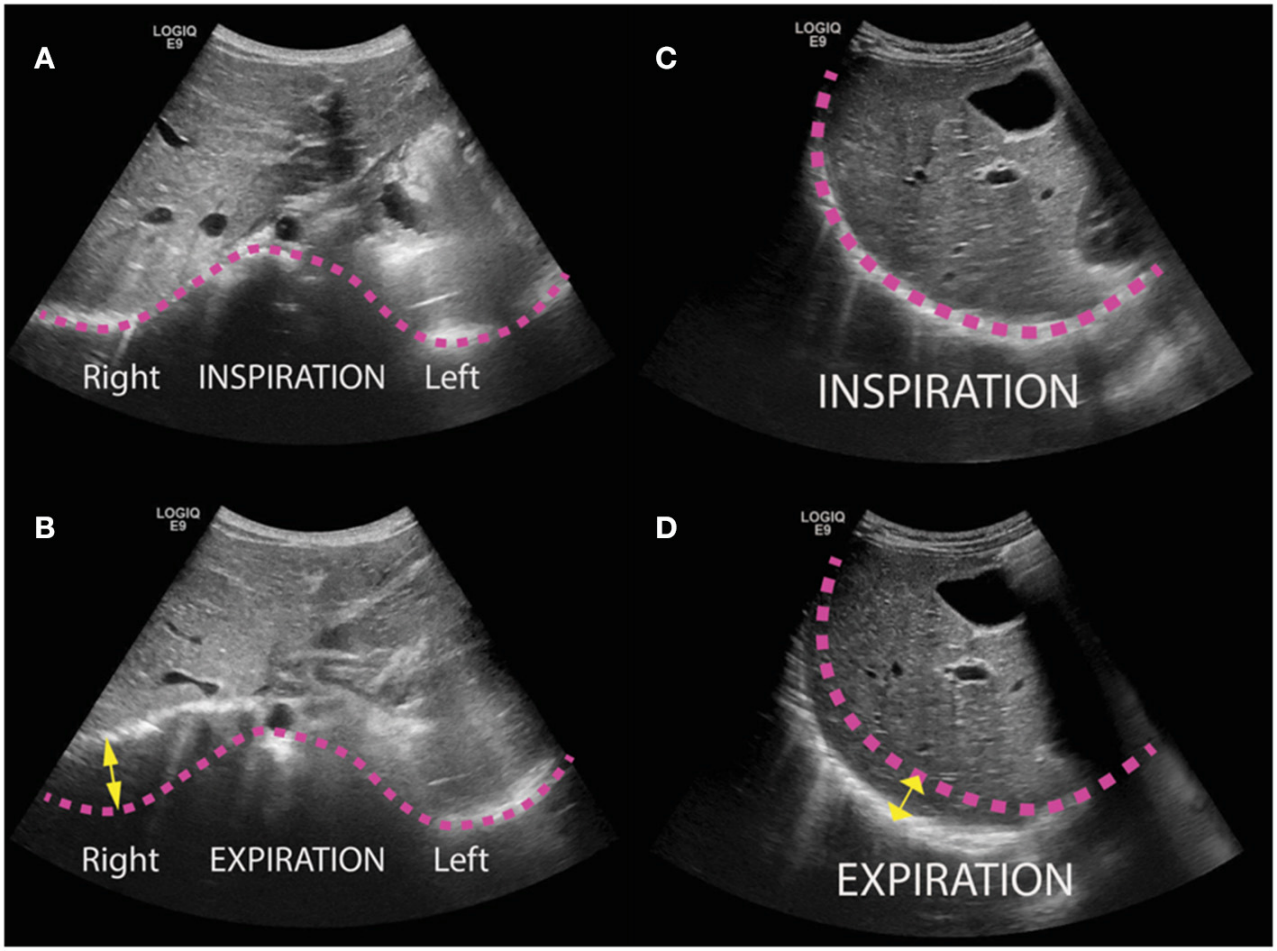
Transthoracic ultrasound images of pediatric patients following repair of congenital diaphragmatic hernia (CDH). Bilateral diaphragms were evaluated in four views (epigastric, anterior, lateral, and posterior) and the greatest craniocaudal excursion of each hemidiaphragm in any view was taken as the maximal excursion. **(A,B)** Illustrative transverse images obtained in a subxyphoid window from a 12-month-old boy status post repair of left CDH with internal oblique muscle flap at inspiration **(A)** and expiration **(B)**. Pink lines trace the contour of the diaphragm in inspiration, and yellow arrow illustrates excursion of the right diaphragm. **(C,D)** Illustrative sagittal images obtained in a 3-year-old girl status post repair with internal oblique muscle flap at inspiration **(C)** and expiration **(D)**.

We conducted a cross-sectional study of patients who were followed routinely after CDH repair at a single children's hospital. Consecutive patients who presented after medium-term follow-up visit between Feb 1 2018 and March 31 2019 underwent transthoracic ultrasound. The ultrasound was performed by a single pediatric radiologist with 7 years of post-fellowship experience. The ultrasound was performed with a General Electric Logiq E9 ultrasound machine using a curved transducer with frequency range of 2–9 MHz (GE Healthcare, Chicago IL). The transducer frequency, gain, and focus depth were manually adjusted for each patient to optimize anatomic detail. The craniocaudal excursion of bilateral diaphragms was assessed in four views during a period of calm breathing. The greatest craniocaudal excursion of each hemidiaphragm in any view was taken as the diaphragmatic excursion (Figure 1). In order to create a metric that could possibly be compared between children of different sizes, the diaphragmatic excursion ratio (DER) was defined as ratio of the repaired-side diaphragmatic excursion (RDE) to the contralateral-side diaphragmatic excursion (CDE) during quiet breathing:

$$\begin{array}{l} DER\;=\;RDE/CDE \end{array}$$

Ultrasounds were performed by a single pediatric ultrasonography technician and interpreted by a single board-certified pediatric radiologist, who were both blinded to the type of repair and the patient's clinical outcome at the time of the study.

Demographic data and details of the perioperative course were collected via retrospective chart review. Patient outcomes after index repair included: age at repair, ventilator days, percentage of patients discharged on supplemental oxygen, days to full feeds post-operatively, percentage of patients on any enteral feeds at discharge, need for gastrostomy, and length of stay. The following outcomes at 1-year post-repair were assessed: readmission for any reason, readmission for pulmonary complication, any enteral feeds. In our practice, prior to 2012 patients with larger defects were treated with mesh patch. However, after 2012 almost all large defects were treated with internal oblique muscle flap. Therefore, in this analysis patients who underwent mesh patch repair and those who underwent repair with internal oblique muscle flap were grouped together as “non-primary repair.” Continuous variables were described using mean and standard deviation, and median and interquartile range. Binary variables were reported as percentages. Statistical comparison of the primary repair group vs. the non-primary repaired group was conducted by Fisher Exact or Kruskal-Wallis tests. A median DER was calculated and clinical outcome variables were analyzed above and below the DER median by Mann-Whitney or Fisher Exact tests. Data were analyzed using Stata Statistical Software: Release 16, College Station, TX. A *P* < 0.05 was considered statistically significant.

## Results

Thirty-eight patients were included [median age at evaluation 24 months, interquartile range (IQR) 11–60]. Thirty-five patients (92%) had left-sided defects, and 22 (58%) had at least part of the liver in the chest. Twenty patients were female (53%). Thirty-one patients (82%) were diagnosed on prenatal ultrasound, with median lung-to-head ratio of 1.2 (IQR 0.9–1.8). No patients were treated with extracorporeal membrane oxygenation. Nine patients underwent primary repair. Twenty-nine patients underwent non-primary repair, 20 with internal oblique muscle flap, and 9 with mesh patch (Table 1). There were no significant differences in the proportion of males, gestational age, proportion of patients with prenatal diagnosis, or proportion of patients with left-sided defects between the two groups. However, only one patient in the primary repair group had any portion of the liver in the chest (11%) compared to 21 (72%) patients in the non-primary repair group (*p* = 0.002).

**Table 1:** Patient demographics.

	**Total**	**Primary repair**	**Non-primary repair**	**Comparison of primary vs**.
	**(*n* = 38)**	**(*n* = 9)**	**(*n* = 29)**	**Non-primary repair (P)**
Male, *n* (%)	20 (53)	6 (67)	14 (48)	0.3
Birth weight, g	3,000 (2,800–3,000)	2,800 (2,500–3,000)	3,000 (2,960–3,000)	0.1
Gestational age, wk	38 (37–39)	39 (37–39)	38 (37–39)	0.3
Prenatal diagnosis, *n* (%)	31 (82)	6 (67)	25 (86)	0.2
Left-sided defect, *n* (%)	35 (92)	9 (100)	26 (90)	NS
Liver up^Φ^, *n* (%)	22 (58)	1 (11)	21 (72)	0.002
**Other malformation**, ***n*****(%)**
Cardiovascular	7 (18)	2 (22)	5 (17)	1.0
Intestinal, anorectal	1 (3)	0 (0)	1 (3)	1.0
Airway/pulmonary	9 (24)	1 (11)	8 (28)	0.4
Skeletal	1 (3)	0 (0)	1 (3)	1.0

*Values listed as number and % or median and interquartile range as appropriate. Statistical comparison via Chi-square test, Fisher Exact or ANOVA*.

Φ *Defined as any portion of the liver in the thoracic cavity*.

There were significant differences in the post-operative courses of patients who underwent different types of repair (Table 2). Patients who underwent primary repair had fewer ventilator days (median 3 vs. 16 days, *p* = 0.0001), shorter hospital lengths of stay (median 23 vs. 69 days, *p* = 0.0002), and were less likely to be discharged on home oxygen (0 vs. 16%, *p* = 0.005). Patients who underwent primary repair were also less likely to be discharged on any enteral feeds (0 vs. 20%, *p* = 0.0003), were less likely to require a G-tube (0 vs. 18%, *p* = 0.0013) and were less likely to require any enteral feeds at 1 year (9 vs. 15%, *p* = 0.01). As a sensitivity analysis, the outcomes for patients who underwent muscle flap and mesh repairs were separated and compared against the outcomes of patients who underwent primary repair (Supplementary Table 1). The results were similar to those seen in the combined non-primary repair group.

**Table 2:** Patient outcomes.

	**Total**	**Primary repair**	**Non-primary repair**	**Comparison of primary vs**.
	**(*n* = 38)**	**(*n* = 9)**	**(*n* = 29)**	**non-primary repair (P)**
Age at repair, days	3 (2–5)	2 (1–3)	3 (3–5)	0.09
Ventilator days	14 (8–20)	3 (2–6)	16 (13–22)	0.0001
Days to full feeds	10 (7–15)	13 (10–19)	10 (7–14)	0.1
Hospital length of stay, days	58 (27–97)	23 (20–24)	69 (44–112)	0.0002
Discharge on O2, *n* (%)	16 (42)	0 (0)	16 (55)	0.005
Any enteral feeds at discharge, *n* (%)	18 (47)	0 (0)	20 (69)	0.0003
G-tube, *n* (%)	18 (47)	0 (0)	18 (62)	0.0013
**Post-operative Year 1**
Readmission for any reason, *n* (%)	15 (39)	1 (11)	14 (48)	0.06
Readmission for pulmonary indication, *n* (%)	7 (18)	0 (0)	7 (24)	0.2
Any enteral feeds at 1 yr, *n* (%)	17 (45)	9 (100)	15 (52)	0.01
Z score - weight for age	−0.6 (−1.4 to 0.0)	−0.5 (−1.5 to 0.6)	−0.7 (−1.3 to −0.1)	0.8
Z score – height for age	−0.4 (−1.1 to 0.6)	−0.1 (−1.4 to 0.9)	−0.5 (−1.0 to 0.3)	0.7

*Mean and SD, or Median and IQR as appropriate. Statistical comparison by Fisher Exact or Kruskal-Wallis tests. Note, in some cases, contingency analysis limited by small groups*.

The median diaphragmatic excursion ratio (DER) of the entire cohort was 0.54 (IQR 0.4–0.7). The median DER of the primary repair group was significantly higher than that of the non-primary repair group (median 0.69 vs. 0.44, *p* = 0.02). When the non-primary repair group was broken out into patients who underwent muscle flap and those who underwent mesh patch repair, the median DER of the primary repair group was significantly higher that that of the muscle flap group (0.69 vs. 0.43, *p* = 0.02). However, there was no difference in the median DER between the primary repair and mesh repair groups or the muscle flap and the mesh repair groups (0.69 vs. 0.59, *p* = 0.1; 0.43 vs. 0.59, *p* = 0.6). When considering all patients together, those with a DER below the median had a significantly longer hospital length of stay following repair (median 77 vs. 28 days, *p* = 0.0007, Table 3) and significantly more ventilator days (median 16 vs. 9 days, *p* = 0.004). Patients with a DER below the median were also significantly more likely to be discharged on home oxygen (68 vs. 16%, *p* = 0.001), and to be using home oxygen at 1 year (32 vs. 5%, *p* = 0.04). Patients with a DER below the median were also less likely to tolerate exclusively oral feeds at discharge (37 vs. 74%, *p* = 0.02) and more likely to have a gastrostomy tube placed in the first year of life (74 vs. 21%, *p* = 0.003). Patients with DER below the median had more readmissions in the first year of life and more respiratory readmissions though neither reached statistical significance. A receiver operator curve was plotted using DER against discharge on O2 (Supplementary Figure 1), and demonstrated an area under the curve of 0.7.

**Table 3:** Relationship between maximal excursion ratio and clinical outcomes.

	**Max excursion**	**Max excursion**	***P***
	**below median**	**above median**	
Length of Stay (days)	77 (58–131)	28 (23–59)	0.0007
Discharge on O2, *n* (%)	13 (68)	3 (16)	0.001
Ventilator days	16 (14–25)	9 (4–17)	0.004
Days to goal feeds	13 (8–16)	10 (7–13)	0.3
Gastrostomy tube in yr 1, *n* (%)	14 (74)	4 (21)	0.003
Exclusively oral feeds at 1 yr, *n* (%)	7 (37)	14 (74)	0.02
Readmission in yr 1, *n* (%)	9 (47)	5 (26)	0.2
Respiratory readmission in yr 1, *n* (%)	5 (26)	2 (11)	0.2
Pt using home 02 at 1 yr, *n* (%)	5 (32)	1 (5)	0.04

*Mean and SD, or median and IQR as appropriate. Statistical Comparison by Mann-Whitney or Fisher Exact tests*.

## Discussion

Our study demonstrated a novel application of transthoracic ultrasound to assess diaphragmatic excursion during quiet breathing in infants and children after CDH repair. Using this routine and non-invasive modality, we then created a simple measurement, the diaphragmatic excursion ratio and found that impaired diaphragmatic motion correlated with adverse respiratory and surgical outcomes. A DER below the median of 0.54 was found to be associated with longer hospital length of stay, more ventilator days, and increased likelihoods of requiring a gastrostomy tube and being discharged on oxygen. To our knowledge, this is the first description of the diaphragmatic excursion ratio and the first described use of two dimensional ultrasound images to evaluate diaphragmatic excursion following CDH repair.

A variety of invasive methods have been described to measure the motion or function of the diaphragm after CDH repair. One technique is the measurement of airway or transdiaphragmatic pressures exerted during crying (13), or elicited by transcutaneous phrenic nerve stimulation (14). While invasive, these techniques have the advantage of directly assessing phrenic nerve function, diaphragmatic conduction, and diaphragmatic function. For example, one group used transcutaneous magnetic stimulation of the phrenic nerve, and an esophageal recording electrode to demonstrate a trend toward prolonged phrenic nerve latency on the side of CDH repair in five patients studied shortly after CDH repair (14). By stimulating one phrenic nerve at end expiration with the mouth occluded, the authors also reported a trend toward reduced maximal inspiratory pressure on the side of repair. Another study using gastro-esophageal pressure sensors to estimate the pressure generated by the diaphragm during quiet breathing and crying, showed that the maximum trans-diaphragmatic pressure generated by patients was decreased immediately after CDH repair and at 1 year but normalized by 5 years (15). A third group compared the maximal excursion of the repaired hemi-diaphragm on ultrasonography with maximal excursion of contralateral diaphragm, and with the diaphragm in age-matched controls in patients at a median age of 16 years (16). The authors found a significantly lower maximal excursion of the repaired diaphragm compared to the unrepaired diaphragm, and compared to age-matched controls, during quiet breathing. However, the authors used M-mode measurements of diaphragmatic excursion that, because it relies on the line of data acquisition being perfectly oblique to the diaphragm, risks underestimating diaphragmatic excursion (17).

Our study provides a noninvasive method to evaluate diaphragm movement after CDH repair. While pulmonary function was not directly measured in this study, we were able to assess diaphragmatic motion by sonographic minimal and maximal excursion of the repaired and unrepaired diaphragm. Furthermore, we created a ratio demonstrating that impaired diaphragmatic motion with a DER below the median was associated with poor respiratory outcomes at neonatal discharge. With relatively simple assessment, we believe that this novel ultrasound measurement can be practically employed on patients after CDH repair with minimal to no risk, is non-invasive, and can provide useful data on respiratory and surgical outcomes.

The existing markers of CDH disease severity, including observed-to-expected Lung area to Head circumference Ratio, likely reflect the impact of the diaphragmatic defect on prenatal lung development. However, evaluation of diaphragmatic excursion and the DER following repair may provide insight into the pathophysiology of lung growth, development, and function after CDH repair.

Our study also evaluated the effect of the type of diaphragmatic repair on respiratory and surgical outcomes. Non-primary repair is a known predictor of poor functional outcome in published studies (3, 18), which is similarly reported in our study. However, in the group of patients who undergo either patch or IOMF repair, there is a wide spectrum in the severity of disease and long-term comorbidities. Therefore, DER can potentially be used to stratify within the non-primary repair group, which constituted 76% of the patients in our cohort.

Overall, birth weight, gestational age, and other associated anomalies were similar between primary and non-primary repair groups. The only statistically significant difference was that a portion of the liver was more likely to be up in the thoracic cavity in non-primary repairs. We speculate that the presence of an intrathoracic liver reflects more severe pulmonary hypoplasia and thus further abnormal lung development. A recent longitudinal study in which serial pulmonary function testing was performed for 3 years after CDH repair demonstrated that the pattern of lung growth following CDH repair differs from that in normal children (19). In general, patients had decreased forced expiratory flow rates and increased functional residual capacities and residual volumes compared to normal. Patterns of lung development over time were also abnormal, with spirometry that suggested lung growth by overdistension of airspaces, rather than the normal rapid increase in alveolar number. Careful study of the movement of optical sensors placed on the chest wall after repair, highlight the widespread changes in respiratory mechanics that occur following CDH repair (20). These include a paradoxical inward inspiratory movement of the pulmonary rib cage on the unrepaired side, and a reduction in the contribution of the abdominal compartment to tidal volumes in some patients. Both major changes suggest a reduction in the effectiveness of the repaired hemidiaphragm.

Our study has several limitations. As a cross-sectional study, evaluation of diaphragmatic motion in individual patients was limited to just one time point, on average about 2 years post-repair. In addition, the relationship between diaphragm movement and respiratory function is not well-defined. Potential confounding factors include altered diaphragmatic and respiratory function following CDH repair, which cannot be controlled in this cohort. Diaphragmatic excursion is only a proxy for diaphragm function and, although we have demonstrated that the DER several years after repair is associated with important short-term outcomes, we have not measured overall respiratory function with conventional pulmonary function tests. It remains unclear if the DER shortly after surgery has the ability to predict adverse outcomes after repair and whether DER changes over time. The next steps include a prospective study with evaluation at multiple points from the early post-operative period into early childhood to determine the relationship between the motion of the repaired and unrepaired diaphragms to pulmonary function and other clinical outcomes.

## Conclusions

Transthoracic ultrasound is a practical technique to evaluate diaphragmatic motion after CDH repair. The diaphragmatic excursion ratio, measured even several years post-operatively, is significantly associated with important short-term outcomes, such as hospital stay, discharge on supplemental oxygen, delay of exclusive oral feeds and need for gastrostomy tube. This likely reflects the important role that diaphragmatic function has on both respiratory function, pulmonary development, and in turn overall patient outcomes. Further prospective studies may help validate this novel ultrasound measurement and may offer prognostic value for children post-CDH repair.

## Data Availability Statement

The raw data supporting the conclusions of this article will be made available by the authors, without undue reservation.

## Ethics Statement

The studies involving human participants were reviewed and approved by University of California San Francisco Institutional Review Board. Written informed consent to participate in this study was provided by the participants' legal guardian/next of kin.

## Author Contributions

JR conceived of the study, collected and analyzed the data, and wrote the draft manuscript. NL collected and analyzed the data. AP conceived of the study, developed the ultrasound technique, interpreted all of the ultrasounds, and edited early and revised versions of the manuscript. AS interpreted the data and drafted and edited the manuscript. LV conceived of the study, interpreted the data, and edited early and revised versions of the manuscript. All authors contributed to the article and approved the submitted version.

## Conflict of Interest

The authors declare that the research was conducted in the absence of any commercial or financial relationships that could be construed as a potential conflict of interest.

## Publisher's Note

All claims expressed in this article are solely those of the authors and do not necessarily represent those of their affiliated organizations, or those of the publisher, the editors and the reviewers. Any product that may be evaluated in this article, or claim that may be made by its manufacturer, is not guaranteed or endorsed by the publisher.
