# Supplementary material for: A Novel Thoracic Ultrasound Measurement After Congenital Diaphragmatic Hernia Repair Identifies Decreased Diaphragmatic Excursion Associated With Adverse Respiratory and Surgical Outcomes
Source: Front Pediatr. 2021 Aug 5;9:707052. doi: 10.3389/fped.2021.707052 (PMC8375432; doi:10.3389/fped.2021.707052)
Supplement: Supplementary file 1 [file Table_1.DOCX]

| Supplemental Table 1. Patient Outcomes by Repair Type | | | | | | |
| --- | --- | --- | --- | --- | --- | --- |
|  | | **Primary Repair**  **(n = 9)** | **Muscle Flap**  **(n = 20)** | **Mesh Repair**  **(n = 9)** | **Comparison of Primary Repair to Muscle Flap (P)** | **Comparison of Primary Repair to Mesh Repair (P)** |
|  | |  |  |  |  |  |
| Age at repair, days | | 2 (1 - 3) | 3 (3 - 5) | 4 (3 - 6) | 0.2 | 0.03* |
| Ventilator days | | 3 (2 - 6) | 15 (13 - 19) | 22 (10 - 30) | 0.0001* | 0.001* |
| Days to full feeds | | 13 (10 - 19) | 10 (7 - 15) | 7 (5 - 12) | 0.2 | 0.09 |
| Hospital length of stay, days | | 23 (20 - 24) | 87 (63 - 124) | 55 (28 - 58) | 0.0001* | 0.01* |
| Discharge on O2, n (%) | | 0 (0) | 12 (60) | 4 (44) | 0.07 | 0.08 |
| Exclusively oral feeds at discharge, n (%) | | 0 (0) | 4 (20) | 5 (56) | 0.3 | 0.03* |
| G-tube, n (%) | | 0 (0) | 14 (70) | 4(44) | 0.0007* | 0.3 |
| **Post-operative Year 1** | |  |  |  |  |  |
|  | Readmission for any reason, n (%) | 1 (11) | 8 (28) | 6 (33) | 0.2 | 0.05* |
|  | Readmission for pulmonary indication, n (%) | 0 (0) | 5 (25) | 2 (22) | 0.2 | 0.5 |
|  | Exclusively oral feeds at 1 yr, n (%) | 9 (100) | 9 (45) | 3 (33) | 0.005* | 0.009* |
|  | Z score - weight for age | -0.5 (-1.5 - -0.6) | -0.4 (-1.2 - 0.0) | -1.3 (-1.4 - -0.9) | 1 | 0.4 |
|  | Z score - height for age | -0.1 (-1.4 - -0.9) | -0.2 (-1.0 - 0.6) | -0.5 (-1.1 - 0.2) | 0.9 | 0.4 |
| Mean and SD, or Median and IQR as appropriate. Statistical comparison by Chi-square or Fisher exact tests. | | | | | | |

Supplemental Table 1
